# Making Research Data Repositories Visible: The re3data.org Registry

**DOI:** 10.1371/journal.pone.0078080

**Published:** 2013-11-04

**Authors:** Heinz Pampel, Paul Vierkant, Frank Scholze, Roland Bertelmann, Maxi Kindling, Jens Klump, Hans-Jürgen Goebelbecker, Jens Gundlach, Peter Schirmbacher, Uwe Dierolf

**Affiliations:** 1 Deutsches GeoForschungsZentrum GFZ, Library and Information Services (LIS), Potsdam, Germany; 2 Humboldt-Universität zu Berlin, Berlin School of Library and Information Science, Berlin, Germany; 3 Karlsruhe Institute of Technology (KIT), KIT Library, Karlsruhe, Germany; University of Cape Town, South Africa

## Abstract

Researchers require infrastructures that ensure a maximum of accessibility, stability and reliability to facilitate working with and sharing of research data. Such infrastructures are being increasingly summarized under the term Research Data Repositories (RDR). The project re3data.org–Registry of Research Data Repositories–has begun to index research data repositories in 2012 and offers researchers, funding organizations, libraries and publishers an overview of the heterogeneous research data repository landscape. In July 2013 re3data.org lists 400 research data repositories and counting. 288 of these are described in detail using the re3data.org vocabulary. Information icons help researchers to easily identify an adequate repository for the storage and reuse of their data. This article describes the heterogeneous RDR landscape and presents a typology of institutional, disciplinary, multidisciplinary and project-specific RDR. Further the article outlines the features of re3data.org, and shows how this registry helps to identify appropriate repositories for storage and search of research data.

## Introduction

In the debate on the access to and reuse of research data we currently witness a dynamic development. Already in 2003 leading research organizations worldwide declared the importance of “Open Access to Knowledge in the Sciences and Humanities” and the relevance of research data as an integral part of scholarly knowledge in the Berlin Declaration [1]. In 2007 the Organization for Economic Co-operation and Development (OECD) published “Principles and Guidelines for Access to Research Data from Public Funding”, which “are intended to promote data access and sharing among researchers” [2]. These are only two early references of many to follow in a widespread and ongoing debate that concerns diverse stakeholders in the scholarly system.

The Royal Society joined this debate through their notable report “Science as an open enterprise” that was published in 2012. In this report, the Royal Society asks scientists to make their data accessible and usable in the sense of an “intelligent openness”: “Where data justify it, scientists should make them available in an appropriate data repository” [3]. This statement is echoed on a political level. The European Commission demands from member states that they pass policies to ensure that “research data that result from publicly funded research become publicly accessible, usable and re-usable through digital e-infrastructures” [4]. The U.S. government went one step further. It obliged its national research agencies to maximize access to digital research data. The Office of Science and Technology Policy (OSTP) directs that “digitally formatted scientific data resulting from unclassified research supported wholly or in part by Federal funding should be stored and publicly accessible to search, retrieve, and analyze” [5]. The European Commission is planning a similar requirement in their 8th Framework Programme HORIZON 2020 [6]. Finding a definition of the term research data that is valid for various scholarly disciplines remains a challenge comparable with the challenge to define a research data repository. What is meant by research data differs according to research methods and the character of research objects in the disciplines. Nevertheless, it is important to examine the concept of research data as research data repositories need to serve different academic and disciplinary communities with their respective concepts of research data. Information infrastructure requirements arise from these contents and user requirements.

In the following, the term research data is defined as digital data being a (descriptive) part or the result of a research process. This process covers all stages of research, ranging from research data generation, which may be in an experiment in the sciences, an empirical study in the social sciences or observations of cultural phenomena, to the publication of research results. Digital research data occur in different data types, levels of aggregation and data formats, informed by the research disciplines and their methods. With regards to the purpose of access for use and re-use of research data, digital research data are of no value without their metadata and proper documentation describing their context and the tools used to create, store, adapt, and analyze them [7].

Data policies increasingly affect scientists and their handling of research data and recommendations and mandates from funders and journals show to be the most effective influence. Data policies require receivers of grants and authors of papers to ensure the accessibility to the data generated within the scope of a project or as the basis of a publication [8]. As an example, a “Data Sharing Policy” requires National Science Foundation (NSF) applicants “to share with other researchers, at no more than incremental cost and within a reasonable time, the primary data, samples, physical collections and other supporting materials created or gathered in the course of work under NSF grants.” [9] The NSF further requires that measures for the implementation of this policy be specified in a “Data Management Plan” [10]. The German Research Foundation (DFG) also expects similar statements concerning the handling of research data in project proposals since 2010, whereby, whenever possible, consideration should be given to “existing standards and data repositories” [11]. A similar requirement can be found in the “Editorial Policies” of scholarly journals publishers, such as Nature Publishing Group: “authors are required to make materials, data and associated protocols promptly available to others without undue qualifications.” Accessibility of the research data is supposed to be achieved “via public repositories” [12].

Although scientists agree with the potential benefit of data sharing for the scientific progress, the majority is still reserved when it comes to practical implementations [13], [14]. Incentives, such as proper citation of data, can help to foster the transition [15]. The integration of data sharing in scholarly communication offers high potential in this respect.

Research data could be made openly accessible via three publication strategies [16]:

- Publication of research data, as an independent information object, through a repository [17].
- Publication of research data with textual documentation as a so-called data paper [18].
- Publication of research data as enrichment of an interpretive text publication (“enriched publication”) [19].

These publication strategies have in common that an information infrastructure is required, which ensures storage and accessibility of the data with a maximum of persistence and reliability. Such infrastructures can include so called data archives, data centers, digital libraries, digital collections and the like. They are collectively summarized under the term Research Data Repositories (RDR).

Up to now there was no comprehensive overview of this infrastructures and their functionalities available. The project re3data.org – Registry of Research Data Repositories change this adverse situation. The project has begun to index research data repositories in 2012 and offers researchers, funding organizations, libraries and publishers a systematic overview of the heterogeneous RDR landscape. In July 2013 re3data.org lists 400 research data repositories. 288 of these are described in detail by a special vocabulary, which was developed in the re3data.org project. In the following we give an overview on the RDR landscape (section 2). Further, we describe the development of the registry, the features of the re3data.org registry and explain how the registry helps to identify appropriate repositories for storage and search of research data (section 3).

### Research Data Repositories Landscape

In 2009 the European Commission concluded that: “The landscape of data repositories across Europe is fairly heterogeneous, but there is a solid basis to develop a coherent strategy to overcome the fragmentation and enable research communities to better manage, use, share and preserve data” [20]. The Commission characterizes the present landscape of information infrastructures appropriately and clearly describes the need for integrated and homogeneous research services.

RDR, and their corresponding services, are characterized by their content (see section 1). They currently store a wide variety of file formats under different conditions for access and reuse. Compared to the storage of research data, activities concerning the standardization of repositories providing research publications are far more established. The Open Archives Initiative (OAI) was set up early to promote the standardization and networking of institutional or disciplinary repositories providing open access to textual information objects, such as research papers (pre- and post-prints), theses and dissertations [21]. In contrast the RDR community lacks a comparable degree of standardization.

Up to now, only a few studies investigated the global landscape of research data repositories. A study on the characteristics of 100 RDR was published by Marcial & Hemminger in 2010 [22]. Schaaf made a similar attempt in 2011 [23]. A look at disciplinary studies shows a large diversity among RDR, even on a disciplinary level, with biomedicine offering an impressive number of RDR, thereby shaping today’s landscape of research data infrastructures. A closer look at the practices in biomedicine shows that many of its requirements can also be seen in other communities. The 2013 edition of the “Molecular Biology Database Collection” (http://www.oxfordjournals.org/nar/database/a/) of the Nucleic Acids Research journal presents 1512 infrastructures where biomedical research data can be deposited [24]. 200 of these infrastructures were closely examined in the scope of the European Life Science Infrastructure for Biological Information Project (ELIXIR). These 200 repositories are operated by about 100 institutions with a total staff of at least 350. A community of several hundred thousand scientists uses these RDR. The annual direct cost for these 200 RDR is approximately 30 million euro [25]. In order to guarantee the sustained operation of the biomedical RDR landscape, ELIXIR has been included in the European Strategy Forum on Research Infrastructures (ESFRI). ESFRI is dedicated to the strategic promotion of research infrastructures that are of central importance for the competitiveness of the European Research Area (ERA). It was already clear from the start of ESFRI in 2004 that research infrastructures are not only to be classified as physical infrastructures, such as research ships or particle accelerators, but also as digital information infrastructures, such as, “electronic archiving systems for scientific publications and databases” [26].

### 2.1 Typology of Research Data Repositories

In the following, we present a typology of RDR. This systematization was developed on the basis of a first analysis of 400 RDR (see section 3). Based on the widespread distinction between institutional and disciplinary repository for scholarly literature [27] we differentiate between institutional, disciplinary, multidisciplinary, and project specific RDR [28]. The following description of RDR outlines the differences between the four repository types. This systematization helps to get an overview of the varying concepts and strategies of infrastructures for the permanent access and re-use of research data.

#### 2.1.1 Institutional research data repositories

Institutional research data repositories are run by an institution such as an university or research institution. On university level the scope is multidisciplinary. Edinburgh DataShare (http://datashare.is.ed.ac.uk) is an example of an institutional RDR from the United Kingdom. This “online digital repository of multi-disciplinary research data sets produced at the University of Edinburgh” [29] is based on the DSpace software framework and was developed in the period 2007 to 2009 [30]. A total of 61 data sets were stored on the repository as of March 2013. Open Data LMU (http://data.ub.uni-muenchen.de) is another example of an institutional RDR from Germany. It was started in 2010 by using the software ePrints and is available for all members of the LMU Munich as a publication platform for research data [31]. This repository stored 35 data sets as of March 2013.

#### 2.1.2 Disciplinary research data repositories

Prominent examples in the field of disciplinary RDR are GenBank or PANGAEA. GenBank (http://www.ncbi.nlm.nih.gov/genbank) started its service in 1982 [32] and defines itself as being a “public database of nucleotide sequences and supporting bibliographic and biological annotation”. The National Center for Biotechnology Information (NCBI) operates this infrastructure, providing information on the nucleotide sequences of more than 250,000 species [33]. PANGAEA – Data Publisher for Earth & Environmental Science (http://www.pangaea.de) is defined as an “[o]pen access library aimed at archiving, publishing and distributing georeferenced data from earth system research” [34]. This RDR is operated by Alfred Wegener Institute for Polar and Marine Research (AWI) and MARUM – Center for Marine Environmental Sciences at the University of Bremen. PANGAEA started with the scope of being a “Paleoclimate Data Center” and was funded by the German Federal Ministry of Education and Research (BMBF) from 1994 to 1997 [35]. In 2011, PANGAEA stored “about half a million data sets from all fields of geosciences” [36].

#### 2.1.3 Multidisciplinary research data repositories

Alongside disciplinary and institutional approaches there are also research data repositories serving multidisciplinary needs. Figshare (http://figshare.com) is a research data repository example that “allows researchers to publish all of their data in a citable, searchable and sharable manner.” [37] Figshare started in 2011 and is operated by Digital Science, a Macmillan Publishers company [38]. A second example is LabArchives (http://www.labarchives.com), a “web-based electronic notebook software”, operated by a private company, that allows scientists “to store, organize, and publish [their] research data” [39].

#### 2.1.4 Project specific research data repositories

The landscape of RDR with a specific focus on the research data resulting from particular research projects is also diverse. The Scientific Drilling Database (SDDB) (http://www.scientificdrilling.org) operated by GFZ German Research Centre for Geosciences can be named as being exemplary here. It provides drilling data that is created in the scope of the Scientific Continental Drilling Program (ICDP) openly reusable [40]. The RDR of the Bern Digital Pantheon Project (http://www.digitalpantheon.ch/repository) is another example. In this RDR high resolution images and visualizations of the Pantheon in Rome are made freely accessible.

Due to the heterogeneous RDR landscape, which is outlined here, it is often difficult for scholars to identify appropriate repositories for the storage of and access to research data.

### 2.2 Need for Research Data Management Services and Tools

Studies on the researchers perspective showing a broad variety of obstacles that affect scholars’ willingness to share their own data. The comprehensive studies by Kuipers & Van der Hoeven [13], Tenopir et al. [14] and the analysis of the ODE project [41] show that the willingness to share data is often strong related to the need of a supportive research data infrastructure. Repositories, which are embedded in scholarly workflows and associated with incentive systems can help to promote data sharing. Tenopir et al., for example, conclude that in a survey of more than 1300 scientists, that “a majority of respondents in almost all disciplines […] would be willing to place at least some of their data into a central data repository with no restrictions”. One of the obstacle to this willingness is the lack of knowledge by scholars on already existing RDR.

At this point re3data.org comes into action. Today, in most academic disciplines it is difficult to gain a comparative overview of existing RDR. Already existing registries like the OpenDOAR – Directory of Open Access Repositories (http://www.opendoar.org) and the ROAR – Registry of Open Access Repositories (http://roar.eprints.org) only contain a small share of research data repositories (<5%) since both registries focus on repositories for scholarly publications. In the last years websites like the OAD – Open Access Directory (http://oad.simmons.edu/oadwiki/Data_repositories) or DataCite (http://www.datacite.org/repolist) started to list RDR. However, all of the mentioned directories only provide rudimentary information about a RDR and their services, such as a short description containing the repository operator, discipline and URL. To overcome the obstacles mentioned in the user surveys [13], [14], [41], it is necessary to offer researchers, funding organizations, libraries and publishers a systematic and easy-to-use overview of RDR. This means, that in contrast to the already existing directories a more detailed description of a RDR is needed if a registry wants to deliver essential information on access to or conditions of reuse of research data. This description would take into account the disciplinary focus of researchers looking for a place to deposit their research data. It would also take into account the researcher’s provisions by providing information on for how long this RDR has been online, how it is funded, does the RDR have a policy and finally who is responsible for the RDR. All of this information is necessary to illustrate the trustworthiness of a RDR.

### re3data.org – Registry of Research Data Repositories

RDR are expected to be of high relevance for researchers in the near future. The scientific and political demand for Open Science [42], [43], including open access to publicly funded research data and results, is bound to fail without trustworthy, persistent and sustainable infrastructures that support researchers to share their research data. Surveys of RDR operators revealed uncertainty about the financial security of these infrastructures for periods longer than five years [13], [44]. Thus, the current strategic research development and funding can be considered as being inadequate.

A vision of how research data will be handled in 2030 was described in a study commissioned by the European Commission in 2010. According to this, researchers will then be able “to find, access and process the data they need”. In addition to this, researchers gathering data will be able to “deposit their data with confidence in reliable repositories”, working on the basis of international standards [45]. A glance at the present heterogeneous RDR landscape shows that the realization of this vision is a central challenge for the scholarly system.

Against the background of the growing demand for data sharing (see section 1) and the heterogeneous landscape of RDR (see section 2), the re3data.org – Registry of Research Data Repositories initiative (http://re3data.org) aims to develop and operate a directory of RDR. The indexed and structured description of RDR of all domains in a web-based registry is the target of this project. Added value is created by adding to these descriptions a quick and easy to use system of information icons that describes elementary features of each RDR.

Project partners in re3data.org are the Library and Information Services department (LIS) of the GFZ German Research Centre for Geosciences, the Berlin School of Library and Information Science at the Humboldt-Universität zu Berlin and the KIT Library at the Karlsruhe Institute of Technology (KIT). The three project partners have a long standing working relationship with the German Initiative for Network Information (DINI). Under the auspices of DINI a policy paper on research data was already published in 2009 [46]. The first phase of the project from January 2012 to December 2013 is funded by the German Research Foundation (DFG).

The main goal of re3data.org is to offer researchers orientation in the heterogeneous landscape of RDR, both in their role as data producers and as data users. Other target groups are research funders and infrastructure facilities, such as data centers and academic libraries. Furthermore, re3data.org aims to make a contribution to establishing a more coherent and integrated “eco system of data repositories” [47]. The registry describes the development of the worldwide RDR scene. This global overview could, for example, be used to identify disciplines in which the RDR landscape is still underdeveloped.

When the project re3data.org started only a few lists of RDR existed and listed only basic information, such as the name of the repository, its operator, and its disciplinary focus. As a first step the project collected and recorded information of approximately 400 infrastructures storing research data by December 2012. Already existing lists of repositories, such as the lists of the OAD – Open Access Directory were used alongside our own investigations. All three project partners independently examined a subset of twenty randomly selected RDR. This first analysis confirmed the impression of an extremely heterogeneous RDR landscape and served as a basis for the creation of a first draft of a schema to describe RDR. The absence of a suitable schema required us to develop a new metadata schema to describe RDR. In a second step the schema was aligned with similar metadata schemes, vocabulary elements were modified, and basic requirements for RDR introduced.

In July 2012 Version 1.0 of the vocabulary for the description of RDR was published together with a documentation [48]. To ensure transparency in the development of the vocabulary, as well as to gain input and acceptance by the community of RDR operators, comments on the vocabulary were not only requested on the project website, but also via emails to various mailing lists. The feedback was very positive and in some cases very elaborate. The project received feedback from reBIND (http://rebind.bgbm.org), DataCite (http://www.datacite.org), and OpenAIREplus (http://www.openaire.eu), among others. All comments were analyzed and discussed by the project team and the suggestions taken into consideration in the revision of the vocabulary leading to version 2.0 of the vocabulary which was published in December 2012 [49]. The vocabulary covers the following aspects:

- general information (e.g. short description of the RDR, content types, keywords),
- responsibilities (e.g. institutions responsible for funding, content or technical issues),
- policies (e.g. policies of the RDR, incl. there URL),
- legal aspects (e.g. licenses of the database and datasets),
- technical standards (e.g. APIs, versioning of datasets, software of the RDR),
- quality standards (e.g. certificates, audit processes),

The current requirements for certification and auditing procedures for RDR were also examined [50]–[54]. It became clear that many of these requirements are not universally applicable to RDR due to the heterogeneous needs in different academic communities and a general lack of RDR standardization. Consequently, re3data.org defined a low entry barrier for RDR to included in the registry. For a repository to be indexed in re3data.org, details on the access to and licensing of the research data are indispensable. If these basic requirements are met the RDR will be indexed and reviewed by the project team. A detailed description of the requirements can be found in the re3data.org vocabulary [49].

**Figure 1:**
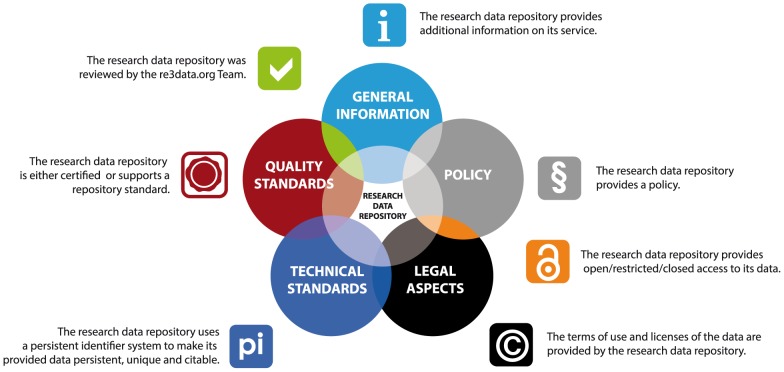
Aspects of a Research Data Repository with the corresponding icons used in re3data.org.

**Figure 2:**
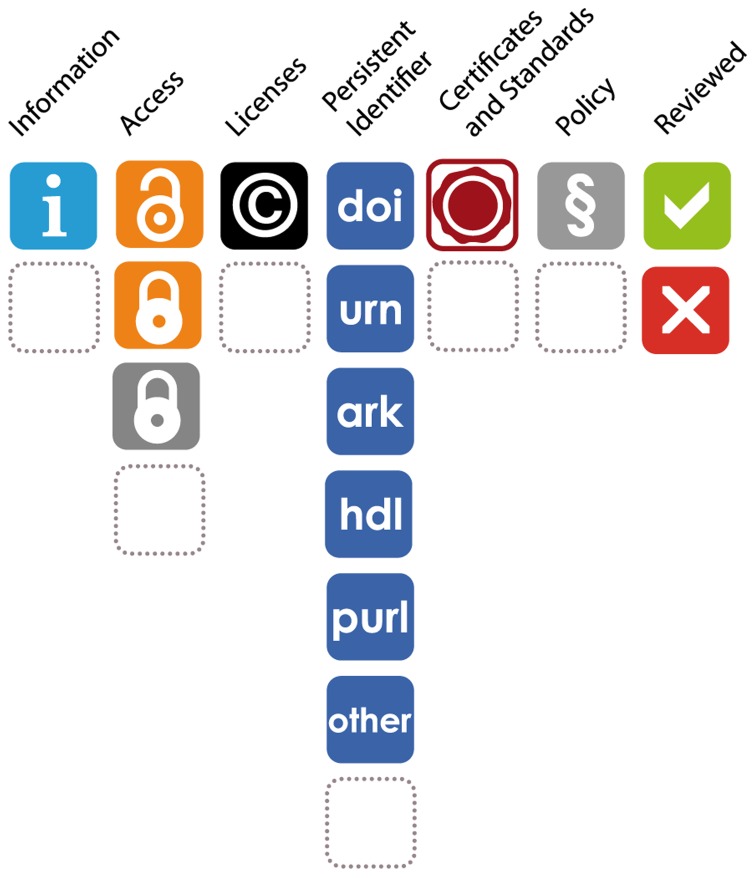
The re3data.org icon system depicting all possible values for each icon.

A set of icons has been developed to show the main characteristics of a repository (Figure 1). The record of an RDR in re3data.org shows up to seven icons (with the respective value) according to the information gathered (Figure 2). This icon system helps users to identify a suitable repository for the storage of their data. In re3data.org researchers can clearly see the terms of access and use of each RDR and other characteristics. The icons and their meaning are explained in figure 1 and 2 and also on the website (http://www.re3data.org/faq/).

However, the icon system is not only useful to the researcher but also to the operators of RDR, allowing them to compare repositories and identify strengths and weaknesses of their own infrastructures. This makes re3data.org also a tool to follow trends in the development of RDRs. Furthermore the icons create an incentive for RDR operating institutions to keep the information about there repository up to date on re3data.org. Our first experiences have shown, that the operators are very interested in a correct and detailed representation of there repositories in re3data.org. Especially the clear presentation of the repositories features via the icon system seems to be an important reason for the RDR operators to keep the information on there infrastructure up to date.

**Figure 3:**
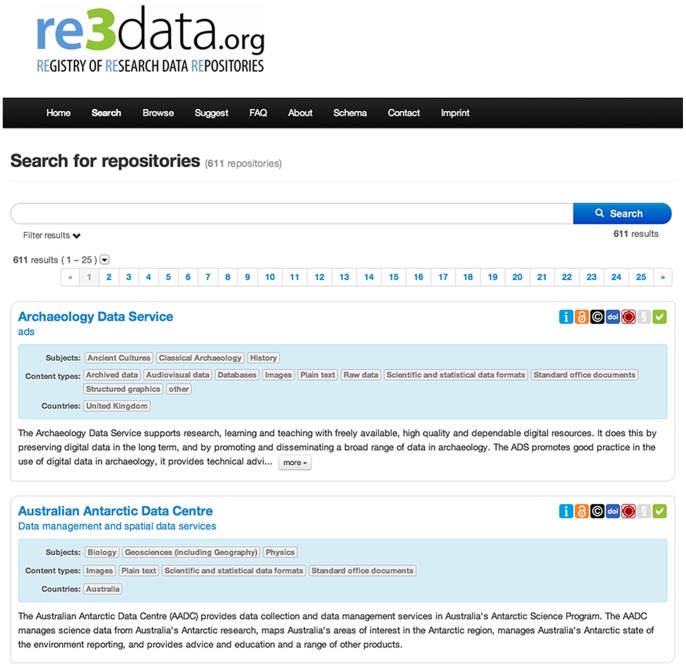
The home page of the re3data.org search.

**Figure 4:**
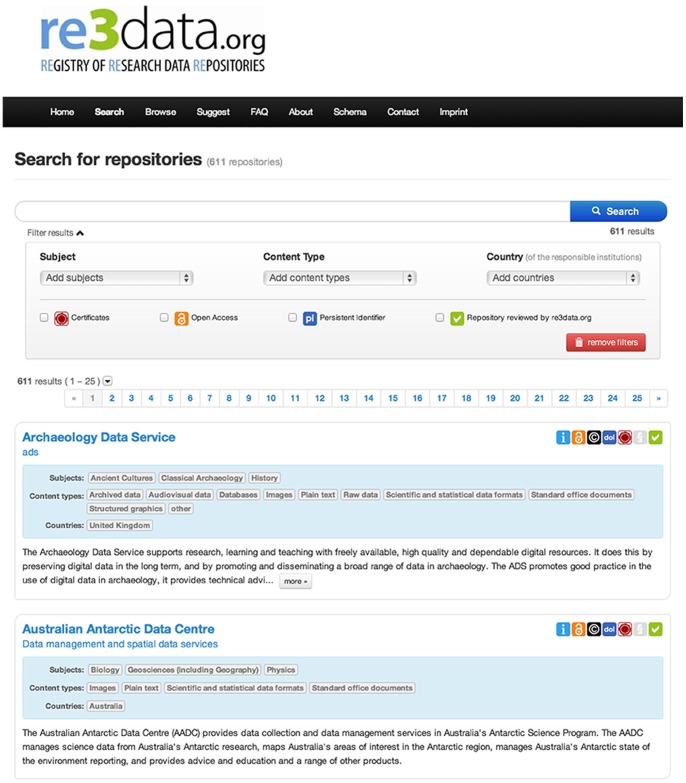
The hit list of a search.

**Figure 5:**
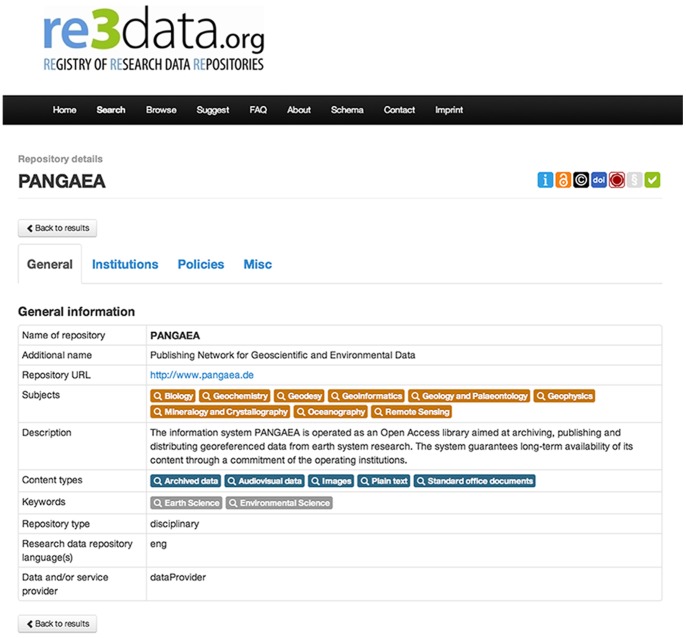
A detailed description of a Research Data Repository.

re3data.org offers two search possibilities: On the one hand a free text search via a simple search box. In this free text search any terms can be searched in the following data base fields: title, additional title, description, keyword, subject, institution, remarks extern (Figure 3). On the other hand the following filters can be used to narrow down the search: subjects, content type, country, certification, open access, persistent identifier and review status. (Figure 4), In the list of results each record includes the name of the RDR, the subjects covered, a brief description of the content and a set of icons visualizing key issues as well as the review status. A comprehensive view of the respective RDR record can be obtained by clicking on the name of the repository (Figure 5).

RDR operators can suggest their infrastructures to be listed in re3data.org via a simple application form. The project team reviews and lists the proposed repositories in the directory. A repository is indexed when the minimum requirements are met, meaning that mode of access to the data and repository as well as the terms of use must be clear explained on the repository pages. Due to the amount of metadata to be collected and the diverging structure of RDR websites the indexing process is time-consuming. Only very few RDR have policies containing essential information on its services, the designated community and the terms of use and in some cases the operators of RDR have to be contacted to obtain this information. Before a new record of a RDR is published in re3data.org all gathered information is reviewed by a second team member. This second step takes at least half as much time as the indexing, however in terms of quality assurance it is absolutely necessary. Based on these first lessons learned, the workflows will be optimized and ways of obtaining feedback from RDR operators improved. Technically a proper workflow management system will cover all steps from ingest to publication of a RDR record (including a persistent identifier).

### Outlook

With her statement “We start the era of open science” Neelie Kroes, EU commissioner for the Digital Agenda (http://ec.europa.eu/digital-agenda), shows that openness will be the paradigm of digital science [42]. The promotion of this development will require a permanent information infrastructure that supports scientists in the sharing of their research data and guarantees access and reuse of research data for the next-generation of researchers.

To provide a persistent registry all re3data.org project partners guarantee the long-term operation of the registry. Based on the feedback from stakeholders re3data.org will go on to develop new features and services in the realm of research data management. Against this setting, a Memorandum of Understanding with DataCite was signed in Spring 2012. DataCite, the initiative for persistent identification of research data, is the result of a project on data publication funded by the German Research Foundation (DFG) in which one re3data.org partner was a consortium member [17]. In the scope of this cooperation, the flow of information between the two partners is particularly important. Consultation with related initiatives like Databib (http://databib.org) is ongoing. In addition to the technical and structural development of the registry, re3data.org and its project partners will continue to contribute to a closer integration and bigger coherence of RDR.

Although re3data.org is still in its starting phase, as of July 2013, 400 RDR have already been indexed in re3data.org out of which 288 have been reviewed. In the next project phase the focus will be on improving the usability and implementing new features. Beyond the development of the registry, the project is dedicated to the networking and standardization of research data depositories. The project strives to make all metadata in the registry available for open use under the Creative Commons license CC0. In doing so, re3data.org contributes to the challenging path to Open Science.
